# Supplementary material for: CuInS2 Nanocrystals Embedded PMMA Composite Films: Adjustment of Polymer Molecule Weights and Application in Remote-Type White LEDs
Source: Nanomaterials (Basel). 2023 Mar 17;13(6):1085. doi: 10.3390/nano13061085 (PMC10058765; doi:10.3390/nano13061085)
Supplement: Supplementary file 1 [file nanomaterials-13-01085-s001.zip › nanomaterials-2288643-supplementary.pdf]

Article

$CuInS_2$ Nanocrystals Embedded PMMA Composite Films: Adjustment of Polymer Molecule Weights and Application in Remote-Type White LEDs

Qingchao Zhou * and Zhongyi Shang

School of Jewelry, West Yunnan University of Applied Sciences, Tengchong 679100, China; 2190235@wyuas.edu.cn
* Correspondence: qczhou@wyuas.edu.cn

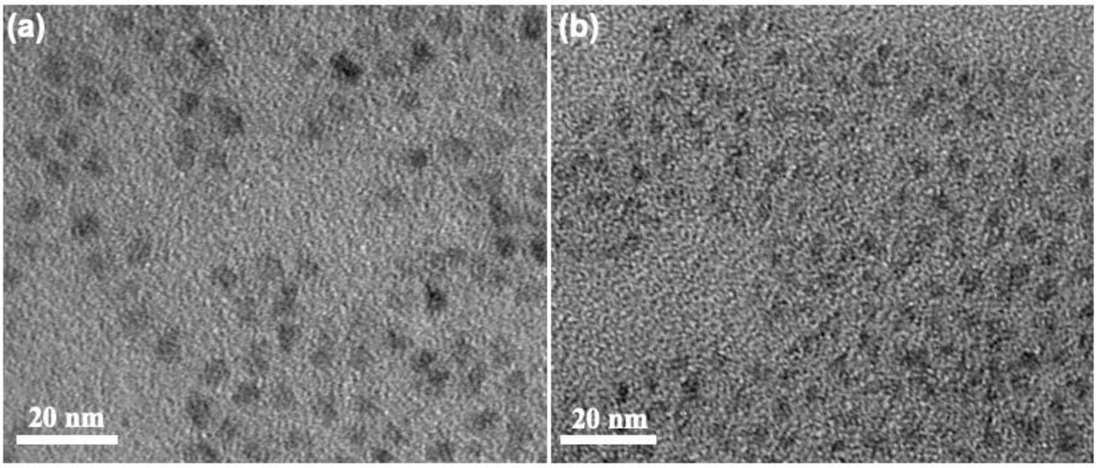

Figure S1. (a) TEM images of green emissive $CuInS_2$ NCs; (b) TEM images of red emissive $CuInS_2$ NCs.

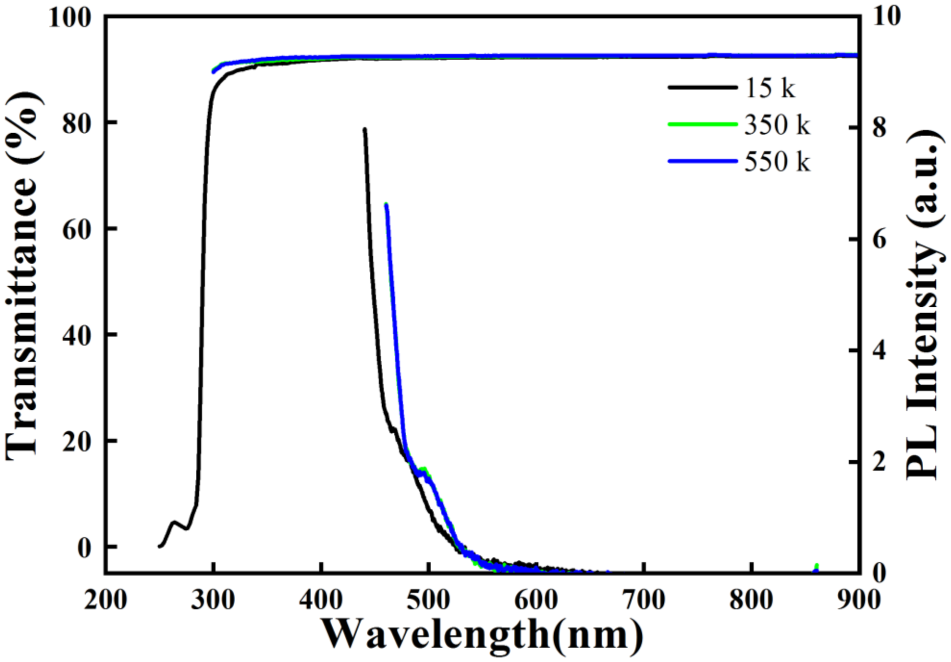

Figure S2. Transmittance and fluorescence spectra of PMMA films with different molecular weights.

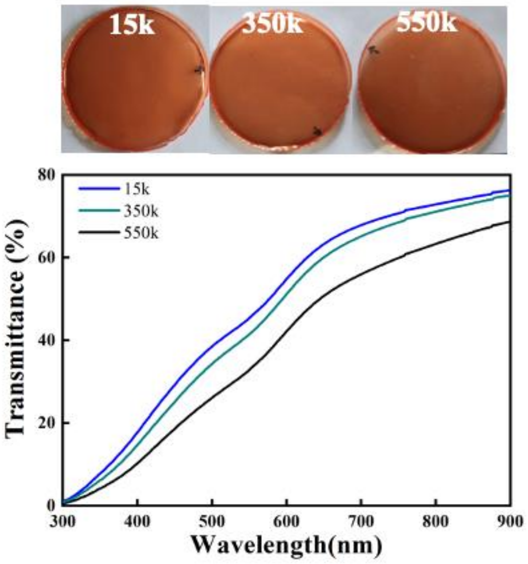

Figure S3. Transmittance spectra of composite films with 5wt% red emissive $CuInS_2$ NCs dispersed in PMMA with different molecular weights.
